# Effects of Low-Intensity Transcranial Magnetic Stimulation in Neuropsychological Development of Pediatric Subjects With Autism Spectrum Disorder: A Longitudinal Retrospective Approach

**DOI:** 10.7759/cureus.76569

**Published:** 2024-12-29

**Authors:** Thonny Augusto Espinosa Mendoza, Alan R Oviedo Lara, Gabriel Henk Jordan, Raúl Sampieri-Cabrera, Luz Erandi Perez Martinez

**Affiliations:** 1 Neuropsychology, Centro de Especialidades Neuropsicológicas Neuroinnova, Guayaquil, ECU; 2 Research, Nibbot International, Mexico City, MEX; 3 Neurosciences, Centro de Especialidades Neuropsicologicas Neuroinnova, Guayaquil, ECU; 4 Foresight, Centro de Ciencias de la Complejidad, Universidad Nacional Autónoma de México, Mexico, MEX; 5 Department of Physiology, Facultad de Medicina, Universidad Nacional Autónoma de México, México, MEX

**Keywords:** autism spectrum disorder (asd), li-tms, low-intensity tms, neurodevelopment disorders, neuro development treatment, neuro-rehabilitation, noninvasive neuromodulation, pediatric rehabilitation, repetitive transcranial magnetic stimulation (rtms), transcranial magnetic stimulation (tms)

## Abstract

Background: Autism spectrum disorder (ASD) is a heterogeneous neurobiological condition characterized by behavioral problems and delayed neurodevelopment. Although transcranial magnetic stimulation (TMS) has been proposed as an alternative treatment for patients with ASD because of its promising benefits in reducing repetitive behaviors and enhancing executive functions, the use of high-intensity pulses (Hi-TMS) appears to be related to the side effects of the therapy. Low-intensity TMS (Li-TMS) has been partially investigated, but it may have clinical effects on ASD and simultaneously increase treatment safety.

Methods: In this study, the effects of combined intervention with Li-TMS and conventional therapies were evaluated in 35 patients from Ecuador (six female and 29 male), aged between three and seven years, with a confirmed diagnosis of ASD. Each subject received conventional therapies twice a week (for four weeks) provided at the research center (psychological, occupational, speech, and neuro-psychomotor therapies) alongside daily Li-TMS sessions at 1 Hz and 9 mT of intensity targeting the left dorsolateral prefrontal cortex (L-DLPFC) for 45 min for four weeks (16 sessions in total). The Battelle Developmental Inventory (BDI), first edition, was applied before and after Li-TMS therapy to evaluate its clinical effects in subjects with ASD. Weekly follow-up assessments and parent questionnaires were administered to identify any adverse events.

Results: In all BDI domains, a significant statistical difference was observed between the pre- and post-intervention averages, supported by extremely low p-values (less than 0.001 in all cases). The personal, social, motor, cognitive, and communicative skills of all the study participants increased after Li-TMS therapy. At the same time, the calculated maturational delay had a significant decrease, suggesting an improvement of ~7.78 months in the neurodevelopment of the ASD subjects. However, age was also found to be a possible cause for these changes in development and maturation. No adverse effects were observed.

Conclusions: Both variants of TMS, Hi-TMS and Li-TMS, have proven to be promising treatments for subjects with ASD, improving social and cognitive abilities. This investigation suggests that the combination of conventional therapies and 16 sessions of Li-TMS as a treatment for individuals with ASD had significant clinical progress, specifically in maturation development according to BDI. In addition, the use of low-intensity magnetic fields may allow for safer pulse delivery in pediatric subjects, as no side effects were reported in this study.

## Introduction

Autism spectrum disorder (ASD) is a heterogeneous neurological condition characterized by behavioral problems and delays in neurodevelopment. Individuals with ASD experience complex symptomatology, including deficits in social interactions and problems with verbal and nonverbal communication. Frequently, these symptoms may also be accompanied by comorbidities, such as intellectual disability, seizures, and anxiety [1,2]. The fifth edition of the Diagnostic and Statistical Manual of Mental Disorders (DSM-5) established that ASD diagnosis requires the identification of persistent deficits in social communication and restrictive and repetitive behavior patterns [3,4]. Furthermore, neurodevelopmental disorders, such as ASD, affect various functional areas, including motor, social, cognitive, and language development [1,5].

Globally, the World Health Organization (WHO) estimates that approximately one in 100 children have ASD [2,5]. In 2016, the National Directorate of Disabilities of the Ministry of Public Health in Ecuador (MSP by its Spanish acronym) reported the existence of 1266 patients diagnosed with ASD [6]. However, the prevalence of ASD-related diagnoses has increased worldwide, including in Ecuador [7]. By analyzing these data together and considering the complexity of ASD diagnosis, it is suspected that there may be underdiagnosed subjects due to the lack of updated diagnostic protocols.

Because conventional pharmacological treatments have increasingly focused on managing comorbidities associated with ASD, recent research has been conducted based on new neuromodulation technologies for the clinical improvement of the core symptoms of autism, offering treatment alternatives to patients [8,9]. Among them is transcranial magnetic stimulation (TMS), which is a noninvasive neuromodulation technique that allows magnetic fields to safely induce transient electrical currents in localized areas of the cerebral cortex where depolarization and neuronal firing are produced. TMS has been studied in clinical trials as a therapeutic tool for ASD, and the results are heterogeneous in terms of stimulation parameters (including areas of stimulation, frequencies, and intensities) and clinical outcomes [10,11]. TMS has recently been classified into two variants: high-intensity transcranial magnetic stimulation (Hi-TMS) and low-intensity transcranial magnetic stimulation (Li-TMS) [12]. The main physical difference between these two types is that the former requires a magnetic strength of repetitive pulses around 1-2 Teslas to induce action potentials, while the second uses a lower magnetic strength range (in the order of milliTeslas (mT) to microTeslas (μT)) to create weak electric fields that modulate brain function without directly causing neuronal depolarization [9,12]. Hence, Li-TMS may also be called subthreshold magnetic stimulation or pulsed electromagnetic fields [12]. This difference also involves variations in the mechanisms of action and device manufacturing. Hi-TMS is characterized by the direct modulation of various neurotransmitters and plays a key role in neuroplasticity by inducing coordinated structural and functional plasticity at excitatory post-synapses. This process is consistent with the LTP of excitatory synaptic transmission [13]. However, Hi-TMS is limited by technical issues (e.g., the need for cooling systems in the devices), safety considerations (headaches and possible seizures), and the fact that the generation of each magnetic pulse produces loud sounds that may result in a challenging scenario when applied to pediatric subjects with ASD [14].

In contrast, Li-TMS uses a wide range of frequencies and patterns at low magnetic intensities that indirectly modulate neuronal excitability, neuroplasticity, and neuronal survival, mainly by acting on calcium-signaling pathways. Although Li-TMS does not induce an action potential, L-type voltage-gated calcium channel activity is increased, and higher levels of Ca^2+^ ions are found intracellularly [15]. These Ca^2+^ level changes have been associated with higher brain-derived neurotrophic factor (BDNF) expression in stimulated neurons [16], which is related to neuroplasticity processes. Although Li-TMS does not alter passive membrane properties such as resting membrane potential and input resistance, it enhances neuronal excitability by inducing a more hyperpolarized action potential threshold and increasing the evoked spike firing frequency compared to sham stimulation [17]. Nonetheless, further studies are needed to understand how weak magnetic fields generate various physiological effects, particularly in individuals with ASD. In addition, the use of lower magnetic fields may also significantly reduce the risk of side effects produced by Hi-TMS related to high magnetic strength. For instance, Li-TMS devices do not generate any sound when magnetic pulses are delivered, reducing the risks of sound-associated adverse events.

Regarding the safety of TMS, different side effects have been reported in clinical trials conducted on infants with ASD. However, because of the heterogeneous information about the stimulation patterns (such as frequency, intensity, number of sessions, and stimulation target), the global assessment of side effects is complicated. The most frequent adverse events reported (when using eight-shaped coils delivering intensities between 80% and 120% of the resting motor threshold, frequencies of 1, 5, 10, and up to 50 Hz and applied over the left or bilateral dorsolateral prefrontal cortex (DLPFC), or the primary motor cortex) include headache, irritability, itching, facial discomfort, sleepiness, pain at the application site, and headedness/dizziness [18]. The 2022 Oxford University TMS Practical Guide highlights the safety and use of Hi-TMS in children. Despite technical and ethical challenges, recent research in both healthy children and those with neurodevelopmental disorders indicates that single- and paired-pulse paradigms are generally safe for children aged two years and older, as long as appropriate hearing protection is used [11].

Recent research has revealed certain neurobiological abnormalities in ASD, such as abnormal brain growth, alterations in the prefrontal cortex (PFC), and atypical neural connectivity. These include local hyperconnectivity and long-distance hypoconnectivity, which affect cognitive and behavioral processes [19]. Additionally, abnormal patterns of electrical activity have been observed, such as an increased frequency of slow brain waves and epileptic seizures associated with dysfunctions in neuronal inhibition and excitation [20].

Several brain areas have been described as involved in mediating the three essential behaviors affected by ASD: stereotypical behavior, social behavior, language, and communication [1]. Neuroimaging studies have revealed atypical brain development, including cortical and subcortical anomalies, as well as functional connectivity. The most commonly affected areas include the frontotemporal and frontoparietal regions, parts of the basal ganglia, and DLPFC [21]. The anterior cingulate cortex was also involved. Its main role is in executive, evaluative, and metacognitive functions, as well as emotional processing. This information is generally processed through connections with the prefrontal and parietal cortices [22,23]. In ASD, involvement of the DLPFC has been frequently proposed, as it is crucial for executive functions such as planning, decision-making, inhibitory control, and working memory [24].

Different investigations have suggested that the application of TMS to the DLPFC at low frequencies improves repetitive behaviors and executive functions in patients with ASD [25]. Some of these studies have reported enhancements in social and communication skills, including increased social reciprocity and improved ability to initiate and maintain social interactions [26]. Advancements have been observed in cognitive skills, such as mental flexibility and planning, as well as in adaptive skills, demonstrating greater independence in their daily activities. These improvements are attributed to a reduction in neuronal hyperexcitability, modulation of circuits involved in behavioral control and executive functions, and facilitation of neural network reorganization [27]. Other researchers have demonstrated that Hi-TMS can significantly reduce repetitive behaviors and enhance executive functions in children and adolescents with ASD [26,28]. Nonetheless, few studies have focused on assessing Li-TMS as a therapeutic tool for subjects with ASD. Makale MT and colleagues recently applied personalized TMS treatment (PrTMS) to subjects with ASD. PrTMS was characterized using low-power magnetic pulses (between 25% and 60% of the resting motor threshold) and spectral electroencephalogram analyses to determine the best frequency pattern for each subject. This approach reported significant reductions in the scale scores of the Autism Spectrum Quotient (ASQ) and Childhood Autism Rating Scale (CARS), as 55% of the subjects analyzed with the ASQ and 44% of the individuals completing the CARS showed a decrease of 15% from the initial values [29]. In addition to the clinical effects of PrTMS, researchers highlighted the safety of this therapy, as no side effects were reported by any of the participants in this study. However, to date, no other reports using much lower magnetic strengths (μT to mT) have been conducted.

In this article, we evaluated the preliminary effects of Li-TMS on neuropsychological development in 35 Ecuadorian children with ASD using a retrospective and longitudinal approach to analyze the changes in the domains included in the Battelle Developmental Inventory (BDI) first edition (which is the edition valid in Ecuador and translated to Spanish). This questionnaire is a comprehensive tool widely used to assess the development of children with ASD between 0 and eight years of age, measuring abilities in areas such as personal/social, adaptive, motor, communication, and cognitive abilities (see Supplementary information, Tables 4-7). The BDI identifies specific delays and strengths, which are then converted into scores to calculate global maturation. This value facilitates the adjustment of interventions and monitoring of the clinical progress of the child by comparing it with chronological age (subtraction of the date of birth from the date of the test). The difference between the two ages (chronological and global maturational) provides the calculated developmental delay of the subject [24]. Although the BDI does not allow specialists to see the underlying neurobiological difficulties, it does show how these difficulties affect neurodevelopment, limiting the acquisition of skills [30].

## Materials and methods

Study design

A retrospective, observational, longitudinal study was conducted to obtain data from clinical records and evaluations using convenience sampling.

Study subjects

Thirty-five patients were enrolled between April 2022 and June 2024, meeting the following inclusion criteria: age between three and seven years, with a confirmed diagnosis of ASD by specialists in psychiatry, neurology, or psychology according to DSM-5 criteria, and a recent EEG study within the past year with a neurological evaluation ruling out seizure risk.

Patients were excluded if they did not meet the age range, lacked a confirmed diagnosis, had previously undergone psychiatric pharmacological treatment, or if the legal guardians refused to sign the informed consent form.

Treatment protocol

The treatment was administered by adapting Hi-TMS protocols from previous systematic reviews and meta-analyses to lower magnetic strength (Li-TMS) [10,25,31-34]. To deliver low-intensity magnetic pulses, a NIBBOT PROFESSIONAL V22-RB4 device (NIBBOT International, San Luis Potosí, Mexico) was used, which is an Li-TMS unit for professional use with a valid ISO 13485 quality management system certification, with a maximum power intensity of 300 Gauss (equivalent to 30 mT). The specific Li-TMS protocol applied in this study used continuous pulses of 9 mT at 1 Hz of frequency, which were applied with a figure-8 coil over the left DLPFC (F3 area), following the international 10-20 system for electrode placement. Li-TMS therapy sought to address emotional and attentional aspects.

All subjects received daily Li-TMS therapy for 45 min, four times per week for four weeks (16 sessions in total). In addition to Li-TMS intervention, conventional therapies were applied twice a week as complementary therapies. These include psychological interventions, language and occupational therapies (for sensory integration and motor skills), and neuropsychomotor circuits. The psychometric evaluation, BDI (first edition in Spanish, validated in Ecuador), was applied before the first Li-TMS session and after the 16th session to compare changes in the neurodevelopmental domains. The results of the baseline assessment were used to confirm the intellectual, language, and motor impairments of each participant to support the ASD diagnosis.

Monitoring and evaluation

Before initiating treatment, the patients and their parents were interviewed to complete a pre-treatment evaluation using the BDI. At the end of the 16th Li-TMS session, the same questionnaire was completed to follow the clinical evolution of the subjects. Additionally, a detailed record of clinical observations and parent reports was collected weekly to evaluate the overall impact of treatment and to identify any possible adverse events (for detailed information, see the weekly parent questionnaire for collection of adverse effects in the appendices section).

For the application of the BDI, it was necessary to prepare the required materials and ensure an appropriate environment to conduct the evaluation, as this is a structured examination that requires an adequate, quiet, and comfortable environment for children. The test includes standardized procedures to apply each of the Battelle items: a) structured evaluation, which consists of assigning specific tasks following a standardized protocol; b) observation, where the abilities of the child are evaluated without direct intervention to obtain an authentic assessment of their behavior; and c) information, which involves collecting details about the behavior of the child through interviews with parents or caregivers since they cannot be directly observed in the session. Initial assessments were carefully conducted, particularly in subjects who were nonverbal or hyperactive. Thus, due to specific communication deficits, the specialists conducted evaluations focusing on recognizing signs of irritability, frustration, aggression, and auto-aggression, as these are common traits in subjects with ASD.

Other application procedures, such as the sequence of items and scoring criteria, allow the qualification of each proposed activity as an objective. In this way, the children’s scores can be compared with the established scales. The age-equivalent or maturational scores corresponded to the direct scores obtained by the child, which can be consulted in the application manual.

Statistical analysis

To assess the normality of the data, a Shapiro-Wilk test was conducted, which obtained a p-value greater than 0.05 (p > 0.05). Therefore, the data were considered normally distributed.

To evaluate the effect of treatment, a paired t-test was performed, which is appropriate for comparing the means of two related measurements within the same group of individuals. This was used to determine whether the differences observed between the pre- and post-intervention measurements of the Battelle scale variables were statistically significant.

In addition to this statistical analysis, the correlation between the pre- and post-treatment values was calculated using Cohen’s d effect to assess the effect size in each domain of the BDI. Finally, with these values, an analysis of covariance (ANCOVA) was performed, considering age as an independent variable and the pre- and post-treatment values as dependent variables.

## Results

The scores of the neuropsychological dimensions included in the BDI were obtained before and after 16 Li-TMS sessions for the 35 subjects (six females and 29 females, Table 1). These were analyzed individually (see Supplementary Figure 2 for individual subject analysis of each domain) and on average. For the group analysis, at the time of the first BDI assessment, the average chronological age of the subjects was 57.751 ± 2.16 months. However, the low scoring of the skills of the BDI domains impacted the global maturation score, which, on average, resulted in an age of 20.44 ± 1.77 months, indicating a maturational delay in their abilities of 37.31 ± 1.95 months (~3.11 years). Table 2 shows the results of the t-test analysis, indicating that higher scores were obtained in all the BDI domains after the subjects received the combined treatment of 16 individual Li-TMS and conventional therapies for one month (average chronological age at the end of the combined treatments was 58.81 ± 2.12 months). The improvement in the domain-specific abilities also resulted in higher global maturational age, increasing 7.7 months from baseline (from 20.44 ± 1.77 to 28.17 ± 2.06 months) and reducing the maturational delay of the subjects in ~0.55 years (average maturational delay at the end of the treatment was 2.55 years).

**Table 1:** Sociodemographic summary

Gender	Count	Mean age (years)	Min age (years)	Max age (years)
Female	6	4.3	3	6
Male	29	4.5	3	7

**Table 2:** Results for each variable of the Battelle scale pre- and post-treatment. For all variables of the Battelle scale, the p-value is <0.001. In all analyzed variables, a significant change is observed between the pre- and post-intervention averages, supported by extremely low p-values (less than 0.001 in all cases).

Variable	Pretreatment mean ± standard error	Posttreatment mean ± standard error
Personal/Social	15.44 ± 1.79	22.83 ± 2.10
Adaptive	25.50 ± 1.75	33.56 ± 2.06
Gross motor skills	22.06 ± 1.76	26.50 ± 2.06
Fine motor skills	24.11 ± 1.67	33.00 ± 2.39
Motricity	21.33 ± 1.57	28.86 ± 2.21
Receptive communication	18.78 ± 2.25	27.81 ± 2.27
Expressive communication	15.00 ± 1.79	21.86 ± 2.24
Communication	15.94 ± 1.80	23.33 ± 2.10
Cognitive	23.92 ± 2.55	33.47 ± 2.59
Global maturation	20.44 ± 1.77	28.17 ± 2.06
Chronological age	57.751 ± 2.16	58.81 ± 2.12
Maturational delay	37.31 ± 1.95	30.64 ± 2.025

**Figure 1:**
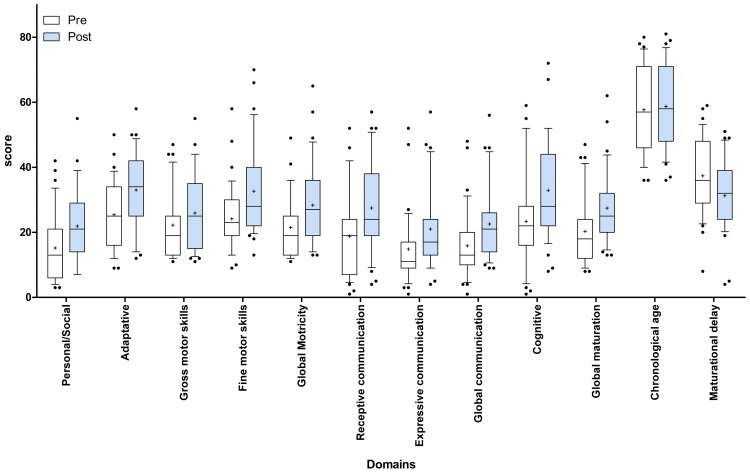
Pre- and post-intervention boxplots for developmental domains. This figure displays boxplots comparing the pre- and post-intervention scores across various developmental domains, including Personal/Social, Adaptive, Gross Motor Skills, Fine Motor Skills, Global Motricity, Receptive Communication, Expressive Communication, Global Communication, Cognitive, Global Maturation, Chronological Age, and Maturational Delay, after one month of Li-TMS therapy. Each domain features two adjacent boxplots, where the left box (in white) represents the scores before the intervention, and the right box (in blue) shows the scores after the intervention. The boxplots illustrate the distribution of scores, the median (central line), and the interquartile range (box). Outliers and mean values are also indicated, providing a clear visual comparison of the changes in each domain due to the intervention. LI-TMS, low-intensity transcranial magnetic stimulation.

Figure 1 compares the changes in each of the different BDI domains before and after one month of Li-TMS treatment and conventional therapies for all subjects. The average of the baseline scores increased for all the evaluated variables except maturational delay, which showed a statistically significant reduction between the pre- and post-intervention averages. The statistical analysis resulted in p-values lower than 0.001 in all cases, indicating that there was a positive statistically significant difference in the abilities of the subjects between the two assessments.

According to the ANCOVA, the age of the subject affected the values obtained in different neuropsychological dimensions included in the BDI, such as personal, adaptive, fine motor skills, and global maturation (Table 3).

**Table 3:** Results for the ANCOVA, correlation, and size effect in each of the evaluated domains pre- and post-treatment with Li-TMS. Values for ANCOVA analysis (p-value) and effect size (Cohen’s d) in each of the assessed neuropsychological dimensions included in the BDI. Pre- and post-values indicate scores before and after Li-TMS treatment. A p-value <0.005 indicated statistical significance in the covariance. Cohen’s d is interpreted as the effect size, where values above 0.8 represent a remarkable effect. The correlation measures the relationship between pre- and post-treatment measurements. ANCOVA, analysis of covariance; Li-TMS, low-intensity transcranial magnetic stimulation.

Dimension	Covariance (p-value)	Cohen's d
Personal/Social pre	>0.005	1.01
Personal/Social post	<0.005	
Adaptive pre	>0.005	1.1
Adaptive post	<0.005	
Gross motor skills pre	>0.005	0.73
Gross motor skills post	>0.005	
Fine motor skills pre	>0.005	0.91
Fine motor skills post	<0.005	
Global motricity pre	>0.005	0.79
Global motricity post	>0.005	
Receptive communication pre	>0.005	0.763
Receptive communication post	>0.005	
Expressive communication pre	>0.005	0.78
Expressive communication post	>0.005	
Global communication pre	>0.005	0.855
Global communication post	>0.005	
Cognitive pre	>0.005	0.749
Cognitive post	>0.005	
Global maturation pre	>0.005	0.822
Global maturation post	<0.005	
Chronological age pre	<0.001	0.998
Chronological age post	<0.001	
Maturational delay pre	<0.001	0.88
Maturational delay post	<0.005	

## Discussion

This study aimed to evaluate the effects of the combination of Li-TMS intervention and conventional therapies (psychological, language, occupational, and neuropsychomotor circuits) on neuropsychological development in pediatric subjects with ASD using the BDI to assess changes in neurodevelopmental skills before and after treatment. Thirty-five subjects (29 male and six female) between 3-7 years (Table 1) were stimulated with Li-TMS protocols focused on the DLPFC, and the findings suggest that this combined treatment had a positive impact in all dimensions of the BDI, which can be interpreted as clinical benefits in the neurodevelopment of the treated patients [35].

All the subjects enrolled in this study had an ASD diagnosis, which was correlated with low baseline scores in the different functional areas analyzed with the BDI associated with intellectual, language, and motor impairments (such as self-help and adaptive skills, motor skills and coordination (motricity), communication, cognitive functioning, and global maturation) (Table 2). The rating of the BDI domains is also used as a predictor of symptom severity in ASD, and lower scores are commonly associated with higher intellectual disability [25]. Therefore, the improvement of the initial BDI rating at the end of the Li-TMS treatment in all the domains (Figure 1) suggests that the combination of conventional ASD approaches (such as psychological, occupational, speech, and neuro-psychomotor therapies) twice a week, with low-frequency and low-intensity magnetic pulses delivered at the DLPFC during 16 sessions (four weeks), enhances specific neurodevelopmental areas of ASD, and therefore, intellectual disability (maturational delay). This can be identified by improvements in a) cognitive abilities, b) focusing and problem resolution, c) language, auditory comprehension, and verbal expression, d) motor control and coordination, e) interaction with family and emotional regulation, e) daily task performance, and f) psychomotor delay. Overall, one of the most important findings of this study is that after one month of this combined intervention, the maturational delay had a statistically significant decrease (Figure 1). According to the interpretation of this inventory, the lower the scores of the skill-related dimensions, the lower the score of global maturation. Consequently, if global maturation is low, the difference between chronological age and global maturation (maturational delay age) will be greater. Here, we identify that one month of Li-TMS + conventional therapies increased the global maturation in 7.73 months (from 20.44 ± 1.77 to 28.17 ± 2.06, Table 2), and this led to a statistically significant decrease in the maturational delay, where the scores obtained at the end of the Li-TMS treatment were lower than the initial values by 5.4 months (from 37.31 ± 1.95 to 30.64 ± 2.025, Table 2), meaning that the neurodevelopment of the evaluated subject is closer to the expected growth according to his/her biological age [31]. The results obtained in this study are statistically significant and not attributable to chance and correlate with other relevant improvements in communication, behavior, and development of the subjects reported by parents in the weekly follow-up reports (including sleep quality without medication, an increase in babbling in non-verbal patients, reduction in irritability, and increase in attention and eye contact during conversation). As per parent reports, at the end of the eighth LI-TMS session (second week of intervention), these effects became more evident. Our results suggest that this combined intervention (Li-TMS + conventional therapies) may have a therapeutic effect on the personal, social, motor, cognitive, communicative, and maturation development of individuals with ASD.

Nonetheless, there were additional variables in this study that could have played a role in the neuropsychological improvement of subjects. The results obtained from the ANCOVA and the effect size observed (Table 3) suggest that the age of the subject has a significant impact on the assessed neuropsychological dimensions. Significant p-values were obtained for chronological age and maturational delay (p < 0.001), indicating that this variable is an important determinant of the observed variance [36]. These dimensions also showed a high effect size (Cohen’s d > 0.8), highlighting the magnitude of this impact. This finding reinforces the idea that age is a correlated factor and possible causal factor in the detected changes in development and maturation, which means that the effect of age could be interpreted as a critical modulator in the progress of the evaluated skills in addition to the applied combined treatment. For instance, domains such as global maturation post-treatment also showed statistical significance (p < 0.005) and a high effect size, suggesting that maturation is narrowly linked to age range, especially when it relates to specific interventions. On the other hand, in domains such as gross motor skills post-treatment, where no significant effects were found (p > 0.005), age could not be the primary factor, but other elements such as basal skills and/or the intensity of the treatment could have greater influence [37]. Altogether, these results support the notion that the age of the subject should be carefully considered when interpreting the effects of Li-TMS treatment and designing personalized interventions, as this influences receptivity and potential improvement in critical domains such as maturation and persona/social skills.

The outcomes of this investigation are similar to those of previous studies in which TMS was evaluated as a therapeutic tool in patients with ASD. For instance, Kaokhieo J and colleagues reported in 2022 that the receptive, expressive, domestic, and community areas of the Vineland Adaptive Behavior Scale increased in ASD subjects after 10 sessions of rTMS at 5 Hz combined with action observation and execution [38]. However, in that report, the stimulation was positioned to target the right inferior frontal gyrus. Barahona-Corrêa et al. found a significant but moderate effect of TMS on social behavior and certain aspects of executive function, as reported by improvements in the Social Responsiveness Scale and the Repetitive Behavior Scale-Revised [25]. Other Hi-TMS studies using bilateral frontal focalization (left and right DLPFC) and low frequencies (1 Hz) have reported improvements in language and social behavior improvements [31,39,40]. Nonetheless, there is important heterogeneity regarding the stimulation patterns (such as intensity and stimulation times), coil stimulation target, and positioning methods [24,41], which complicates a deeper comparison without findings. In this study, the left DLPFC was targeted as it has an important role in social cognition, work memory, and executive control, which are dimensions frequently altered in ASD [32]. Brain activity alterations in alpha and gamma oscillations in the DLPFC are related to functional connectivity deficits in ASD patients. Synchronization by rTMS to specific brain oscillations (i.e., alpha oscillations) may restore normal connectivity patterns. Previous research has demonstrated that high-frequency rTMS applied to the left DLPFC is safe and may reduce clinical manifestations by influencing the connectivity of the cortical networks associated with social behavior and emotional regulation [33,34].

In addition, the effects of Li-TMS were investigated. It was reported that the application of repetitive low-intensity magnetic fields (250-600 mT) via a personalized spectral EEG analysis-designed TMS treatment (PrTMS) to subjects with ASD improved the psychometric scores (particularly cognitive function) in the ASQ and the CARS by reducing more than 15% of the initial score in 44% of the subjects [30].

Although Hi-TMS has already been investigated as a therapeutic tool in ASD subjects, with promising improvements in behavioral deficits (such as repetitive and stereotypical) and verbal social [10,18], some mild and transient side effects (such as facial discomfort, irritability, pain at the application site, and headedness or dizziness) remain prevalent in at least 25% of subjects [18]. In this study, weekly clinical evaluations and parent reports were conducted during the treatment phase to monitor the progress of the participants and rule out possible adverse effects (see supplementary information for reference of the questionnaire applied to the parents). These evaluations allowed for the adjustment of the therapeutic approach according to individual needs, ensuring safe and effective treatment [42]. In contrast to Hi-TMS research, the results of this investigation suggest that Li-TMS is a much safer therapeutic tool for ASD, as none of the most common side effects of Hi-TMS (normally using 300-1100 mT) [18,30] have been reported with the application of low-intensity magnetic pulses in the frontal cortex (9 mT).

The limitations of this study should be considered when interpreting the results and orienting future research. Although this retrospective and longitudinal approach allowed the reporting of preliminary data in an accessible manner considering ethical and logistic limitations when working with pediatric populations, it did not establish causal relationships between the Li-TMS intervention and the results. In addition, the absence of placebo and control groups prevented precise evaluation of the efficacy of Li-TMS treatment in comparison with other therapies or basal conditions. Furthermore, the lack of uniformity in the application of complementary therapies triggers possible confusion in the observed effects, complicating the exclusive attribution of neuropsychological development improvement to the main intervention (Li-TMS). In addition, the small sample size limits the generalization of these findings and increases the susceptibility to high standard errors, specifically when analyzing sub-categories. Similarly, some variables that may play an important role in the subject’s neuroplasticity, neuropsychological development, and response to treatment (such as the age of the participant, comorbidities, and previous treatments) were not adequately controlled. Additionally, research on molecular and functional brain activity is needed to determine the biological mechanisms of low-intensity magnetic pulses in the stimulated area and to evaluate the structural impact of these effects. Although assessment instruments such as the Battelle Developmental Inventory have been used to evaluate the neuropsychological skills of individuals, these evaluations are insufficient to capture specific and multidimensional changes. It is necessary to include other tests, such as CUMANIN or ENI-2, which allow a more precise assessment of the psychomotor and cognitive skills of the subject. Finally, exclusive targeting to the left DLPFC does not consider possible effects on other cortical areas or the impact in the short, medium, and long term. Long-term studies are required to evaluate if additional stimulation sessions lead to higher rates of improvement, validate the duration of the effects, and assess the safety of Li-TMS therapy.

## Conclusions

In this observational retrospective, 35 Ecuadorian children with ASD between 3-7 years of age received a combined intervention of Li-TMS (16 sessions over four weeks) and conventional therapies (twice a week for four weeks) and showed therapeutic effects observed by improvement of the scores of the domains included in the Battelle Developmental Inventory after treatment. These effects were clinically observed by improvements in the personal, social, motor, cognitive, and communicative domains, and particularly by the increase in maturational development skills, potentially reaching chronological age, suggesting an overall effect on neurodevelopment in children with ASD. However, the improvements could not only be attributable to this combined treatment but also to other variables (such as age), which may also play a role in the improvement of the neuropsychological features of the children. Although these enhancements may be useful for focusing on or adjusting the treatment, particularly in the domains that showed a lower response (gross motor skills), the long-term effects on the maintenance of neurodevelopmental maturation remain unknown, leaving potential questions for future research.

TMS in both its variants, Hi-TMS and Li-TMS, has been demonstrated to be a promising neuromodulation tool for subjects with ASD. However, it seems that the use of low-intensity magnetic fields may allow safer pulse delivery in pediatric subjects because of the lower adverse event report rate compared to Hi-TMS. Furthermore, Li-TMS should be considered as a complementary treatment to other conventional ASD therapies as it may potentiate their clinical effects (or vice versa), particularly to enhance the neurodevelopment of ASD subjects at an early age. Nonetheless, additional experimental investigations with rigorous variable setup and control groups are required to assess the individual effects of Li-TMS in comparison to other therapies.

## Figures and Tables

**Figure 2:**
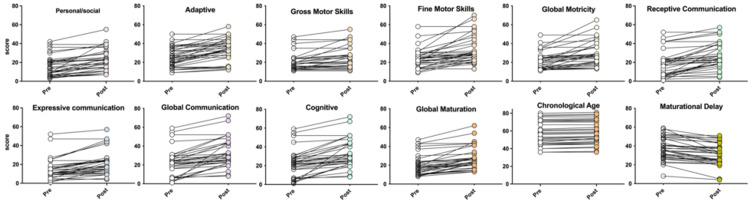
Changes in the neuropsychologic dimensions evaluated with the Battelle Developmental Inventory pre- and post-intervention with Li-TMS therapy. Comparison of the scores obtained pre- and post-Li-TMS intervention in the different domains of the neuropsychologic development evaluated with BDI. Each panel depicts a specific dimension: *Personal/Social, Adaptive, Gross motor skills, Fine motor skills, Global motricity, Receptive communication, Expressive communication, Global communication, Cognitive, Global Maturation, Chronological age, and Maturational delay. *The lines connect the individual scores for each participant, reflecting the evolution after the Li-TMS intervention. The results here suggest improvement in multiple areas. However, interindividual variations are observed. LI-TMS, low-intensity transcranial magnetic stimulation.

## Appendices

Weekly parent questionnaire for collection of adverse effects 

Please answer the following questions:

1. Has the patient reported any headaches in the past week?

2. Have the patient reported or have you noticed discomfort, pain, or itching at the stimulation area?

3. Have you noticed any action out of the ordinary in your child?

4. Has the child's sleep quality changed in the past week?

5. Have you noticed any increase in irritability in the past week?

Please mark with an "X" if any of the following events were reported by the subject (or detected by you) in the last week)

- Headache

- Irritability

- Itching

- Facial discomfort

- Sleepy

- Pain at the application site

- Headedness/dizziness

- Trouble concentrating

- Fatigue

- Stiff neck

- Mild scalp irritation

- Neck pain

- Nausea

- Being more emotional

- Transient muscle spasms

- Seizure

- Other (please specify): _______________

**Table 4:** Battelle Developmental Inventory - General scoring

	Subcategory	Direct Score	Centile score	Typical score Ez T Cl ECN	Equivalent age in months (Tables N-53 N-55)	Z	-5		-4		-3		-2		-1		0		+1		+2		+3		+4		+5	
						T	0		10		20		30		40		50		60		70		80		90		100	
						Cl	25		40		55		70		85		100		115		130		145		160		175	
						ECN	-55		-34		-13		8		29		50		71		92		113		134		155	
PERSONAL/SOCIAL	Interaction with adults																											
	Expression of feelings/affect																											
	Self-concept																											
	Interaction with peers																											
	Collaboration																											
	Social role																											
	TOTAL PERSONAL/SOCIAL																											
ADAPTIVE	Attention																											
	Nutrition																											
	Dress																											
	Personal responsibility																											
	Chores																											
	TOTAL ADAPTIVE																											
MOTOR	Muscular control																											
	Body coordination																											
	Locomotion																											
	Gross motor score																											
	Fine motor score																											
	Perceptive motor score																											
	Fine motor score																											
	TOTAL MOTOR																											
COMMUNICATION	Receptive																											
	Expressive																											
	TOTAL COMMUNICATION																											
COGNITION	Perceptual discrimination																											
	Memory																											
	Reasoning and scholarly habits																											
	Conceptual development																											
	TOTAL COGNITION																											
	TOTAL SCORE																											

**Table 5:** Battelle Developmental Inventory - Subcategory Collaboration Scoring

Subcategory: Collaboration									
Age (months)	Item	Behavior	Score						Observations
18-23	PS 62	Follows everyday life rules.	2		1		0		
24-35	PS 63	Follows the rules given by an adult.	2		1		0		
48-59	PS 64	Obey adult commands.	2		1		0		
60-71	PS 65	Obeys class rules and commands.	2		1		0		
	PS 66	Waits for their turn to get the adult's attention.	2		1		0		
	PS 67	Seeks alternatives to solve a problem.	2		1		0		
	PS 68	Faces teasing and quarrels.	2		1		0		
72-83	PS 69	Participates in new situations.	2		1		0		
84-95	PS 70	Uses the adult to defend themselves.	2		1		0		
	PS 71	Confronts peer aggression.	2		1		0		
				+			=		Subcategory scale

**Table 6:** Batelle Developmental Inventory - Subcategory Social Role Scoring

Subcategory: Social Role									
Age (months)	Item	Behavior	Score						Observations
24-35	PS 72	Plays by taking on adult roles.	2		1		0		
	PS 73	Acts out a role.	2		1		0		
36-47	PS 74	Knows whether they are a boy or a girl.	2		1		0		
	PS 75	Recognizes the differences between men and women.	2		1		0		
48-59	PS 76	Recognizes facial expressions of feelings.	2		1		0		
	PS 77	Plays by taking on the role of an adult.	2		1		0		
	PS 78	Helps when necessary.	2		1		0		
	PS 79	Respects others' belongings.	2		1		0		
	PS 80	Asks for permission to use someone else's things.	2		1		0		
60-71	PS 81	Recognizes others' feelings.	2		1		0		
	PS 82	Distinguishes acceptable behaviors from unacceptable ones.	2		1		0		
72-83	PS 83	Distinguishes present and future roles.	2		1		0		
84-95	PS 84	Demonstrates responsibility.	2		1		0		
	PS 85	Recognizes the responsibility for their errors	2		1		0		
				+				=	Subcategory score

**Table 7:** Battelle Developmental Inventory - Subcategory Attention Scoring

Subcategory: Attention									
Age (months)	Item	Behavior	Score						Observations
0-5	A1	Directs gaze toward a light source.	2		1		0		
	A2	Looks at an object for 5 seconds.	2		1		0		
	A3	Pays attention to a continuous sound.	2		1		0		
06-nov	A4	Follows a light with gaze in a 180° arc.	2		1		0		
	A5	Follows a light with gaze in vertical movement.	2		1		0		
	A6	Engages without seeking attention.	2		1		0		
dic-17	A7	Looks at or points to a drawing.	2		1		0		
18-23	A8	Concentrates on their own task.	2		1		0		
36-47	A9	Pays attention.	2		1		0		
	A10	Pays attention while in a group.	2		1		0		
				+				=	Subcategory score

**Table 8:** Battelle Developmental Inventory - Subcategory Food Scoring

Subcategory: Food									
Age (months)	Item	Behavior	Score						Observations
0-5	A11	Reacts anticipatorily to food.	2		1		0		
	A12	Eats puree with a spoon.	2		1		0		
06-nov	A13	Eats semi-solids.	2		1		0		
	A14	Holds their bottle.	2		1		0		
	A15	Drinks from a cup with help.	2		1		0		
	A16	Eats pieces of food.	2		1		0		
dic-17	A17	Begins to use a spoon or fork to eat.	2		1		0		
	A18	Requests food or drink with words or gestures.	2		1		0		
18-23	A19	Drinks from a cup or glass without help.	2		1		0		
	A20	Uses a spoon or fork.	2		1		0		
	A21	Distinguishes edible from inedible.	2		1		0		
24-35	A22	Gets water from the tap	2		1		0		
36-47	A23	Serves food.	2		1		0		
72-83	A24	Uses a knife.	2		1		0		
				+				=	Subcategory score
