# Correction: Effects of Low-Intensity Transcranial Magnetic Stimulation in Neuropsychological Development of Pediatric Subjects With Autism Spectrum Disorder: A Longitudinal Retrospective Approach

**DOI:** 10.7759/cureus.c241

**Published:** 2025-08-13

**Authors:** Thonny Augusto Espinosa Mendoza, Alan R Oviedo Lara, Gabriel Henk Jordan, Raúl Sampieri-Cabrera, Luz Erandi Perez Martinez

**Affiliations:** 1 Neuropsychology, Centro de Especialidades Neuropsicológicas Neuroinnova, Guayaquil, ECU; 2 Research, Nibbot International, Mexico City, MEX; 3 Neurosciences, Centro de Especialidades Neuropsicologicas Neuroinnova, Guayaquil, ECU; 4 Foresight, Centro de Ciencias de la Complejidad, Universidad Nacional Autónoma de México, Mexico, MEX; 5 Department of Physiology, Facultad de Medicina, Universidad Nacional Autónoma de México, México, MEX

The Conflicts of Interest statement in this article has been corrected to disclose the following conflict of interest:

Financial relationships: Alan R. Oviedo Lara and Luz Erandi Perez Martinez declare employment from Nibbot International.

